# Transcriptional Identification of Related Proteins in the Immune System of the Crayfish *Procambarus clarkii*

**DOI:** 10.3390/ht7030026

**Published:** 2018-09-12

**Authors:** Gabina Calderón-Rosete, Juan Antonio González-Barrios, Manuel Lara-Lozano, Celia Piña-Leyva, Leonardo Rodríguez-Sosa

**Affiliations:** 1 Departamento de Fisiología, Facultad de Medicina, Universidad Nacional Autónoma de México, Ciudad Universitaria, C. P. 04510 Ciudad de México, Mexico; gcalderonrosete@yahoo.com.mx; 2 Laboratorio de Medicina Genómica, Hospital Regional “Primero de Octubre” ISSSTE, Av. Instituto Politécnico Nacional 1669, 07300 Ciudad de México, Mexico; jantgonzalez69@gmail.com; 3 Departamento de Fisiología, Biofísica y Neurociencias, Centro de Investigación y Estudios Avanzados, Av. Instituto Politécnico Nacional 2508, San Pedro Zacatenco, 07360 Ciudad de México, Mexico; manuellara.mvz@gmail.com (M.L.-L.); plceliaqfb62812@gmail.com (C.P.-L.)

**Keywords:** crayfish, *Procambarus clarkii*, transcriptome, immunity, crustacean

## Abstract

The freshwater crayfish *Procambarus clarkii* is an animal model employed for physiological and immunological studies and is also of great economic importance in aquaculture. Although it is a species of easy husbandry, a high percentage of its production is lost annually as a result of infectious diseases. Currently, genetic information about the immune system of crustaceans is limited. Therefore, we used the abdominal nerve cord from *P. clarkii* to obtain its transcriptome using Next Generation Sequencing (NGS) to identify proteins that participate in the immune system. The reads were assembled de novo and consensus sequences with more than 3000 nucleotides were selected for analysis. The transcripts of the sequences of RNA were edited for annotation and sent to the GenBank database of the National Center for Biotechnology Information (NCBI). We made a list of accession numbers of the sequences which were organized by the putative role of the immune system pathway in which they participate. In this work, we report on 80 proteins identified from the transcriptome of crayfish related to the immune system, 74 of them being the first reported for *P. clarkii*. We hope that the knowledge of these sequences will contribute significantly to the development of future studies of the immune system in crustaceans.

## 1. Introduction

Decapod crustaceans show a high capacity for living in diverse environments. The freshwater crayfish *Procambarus clarkii* is a species widely used in physiological studies in diverse laboratories and as a biomarker in contaminated ecosystems [1]; it can even be a predator [2,3], which suggests that it is an adaptable organism with an efficient immune system. However, during its husbandry, the crayfish is impacted by diseases that cause considerable losses to farmers.

*Procambarus clarkii* has been considered one of the three viable species as a model of crustaceans [4]. It is also one of the most farmed shrimp species for human consumption, with production estimated in 2014 by the Food and Agriculture Organization at 6.9 million tons of crustaceans (USD 36.2 billion), of which 723,288 tons corresponded to the cultivation of *P. clarkii* [5]. Because of the economic value of its production worldwide, it is important to limit and effectively control infections that can diminish its profitability.

The interrelation of the neuroendrocrine system with the immune system forms an essential regulatory network in the homeostasis of both vertebrate and invertebrate [6] alterations in the immune system, triggering effects that are essentially related to changes in mRNA expression [7], therefore, the study of the immune system of these invertebrates and knowledge of the molecular mechanisms that constitute it are of great importance. However, the genomic data available are limited for crustaceans in general; for example, some projects such as the i5K [8], which plans to obtain the genomes of 5000 species of insects and other arthropods, has so far only included three species of Decapoda (*Caridina multidentata*, *Marsupenaeus japonicus*, and *Penaeus monodon*) [9]. The first crustacean genome sequenced was *Daphnia pulex*, which made it easier to identify the components of its immune system [10,11]. Since then, another 18 genomes of crustaceans have been sequenced, although only four are decapods: *Procambarus virginalis* (BioProject PRJNA356499) *Caridina multidentata* (BioProject PRJDB4543) *Marsupenaeus japonicus* (BioProject PRJNA387410), and *Penaeus monodon* (BioProject PRJNA387410).

Recently, several authors have published the transcriptome sequences of different tissues from *P. clarkii* in the GenBank database, but so far (June 2018), there have only been 801 protein sequences with annotation for this species.

The growing number of raw sequences deposited in the databanks contrasts with the few sequences with annotation, and so limits the research with automatized identification methods such as in the immune system of crustaceans [12].

A few years ago, the characterization of a protein required molecular biology processes such as polymerase chain reaction (PCR), cloning, and Sanger’s sequencing, which allowed obtaining a limited number of sequences at once. The Next Generation Sequencing (NGS) technologies allow us to obtain thousands of high-quality sequences in any species with considerable advantages in the reduction of time and costs [13].

We applied NGS to obtain the transcriptome of the abdominal ganglionic cord of the crayfish *P. clarkii*. Until now, the eyestalk and the identified genes of the neuroendocrine system explicitly produced in this structure has been the only anatomical region of the nervous system to be analyzed at the molecular level [14]. Therefore, the analysis of the abdominal ganglionic chain that we present in this work is a complement to integrate the knowledge of the nervous system of this species, with the purpose of identifying specific sequences to facilitate the design of molecular tools for its particular study. During the bioinformatic analysis, we identified several proteins that participate in the immune system; we performed a manual Basic Local Alignment Search Tool (BLAST) search for the obtained transcripts and identified several molecules involved in the immunity system of invertebrates.

The major part of the molecules identified have not been previously reported for *P. clarkii*, and some of them have also not been reported for other decapod species. With this work, we aim to contribute to the future study of the immune molecular mechanisms in this species, since knowing these sequences will allow the design of specific molecular tools for their characterization and study.

## 2. Materials and Methods

We used four adult *P. clarkii* crayfish of both sexes in their intermolt stage. The animals were acquired from a local provider and kept in the laboratory in aerated water containers for two weeks prior to the experiments, with a program of 12:12 h of light-dark cycles and fed with carrots and dried fish. The care and handling of the animals during the experimentation procedures were carried out according to the policies of the Society for Neuroscience [15]. This study was approved by the Ethic Commission of the Faculty of Medicine, UNAM with code 86/2016.

Total RNA was extracted from the abdominal nerve cord by using the Trizol LS reagent (Catalog number SKU 10296028, Invitrogen Co., Carlsbad, CA, USA) in accordance with the manufacturer’s protocols. Briefly, Trizol solubilizes the biological material after the addition of chloroform (Catalog number P3803, Sigma-Aldrich, St Louis, MO, USA) producing two phases: the upper containing RNA and the lower containing proteins. The upper phase was transferred to a new tube, the RNA was precipitated with isopropanol (Catalog number I9516, Sigma-Aldrich, St. Louis, MO, USA) and collected by centrifugation, after which the pellet was washed with 75% ethanol (Catalog number E7023, Sigma Aldrich Co., St. Louis, MO, USA). Then, after the ethanol was carefully removed, the pellet was resuspended in RNase free H_2_O and stored at −80 °C. We used 5 μg of total RNA to obtain the cDNA libraries according to the manufacturer’s protocols for an Illumina TruSeq RNA Library Preparation Kit v2 (Catalog number RS-122-2001, Illumina, San Diego, CA, USA). The Illumina paired end protocol 150 bp sequencing was completed. The library obtained was sequenced using the MySeq Reagent kit v3 (Catalog number MS-102-3001) system following the manufacturer’s protocols to obtain the abdominal nerve cord transcriptome.

The Illumina system displayed 40,867,860 raw reads; we used support from the Galaxy Web Portal to execute a de novo assembly with Trinity software [16,17,18]. According to information on the Galaxy portal, “Trinity is a de novo transcript assembler that uses RNA-seq data as input. This tool runs all Trinity commands Inchworm, Chrysalis, and Butterfly in a single pass. This version of Trinity runs on Bridges at the Pittsburgh Supercomputing Center using a version of Trinity 2.2.0 optimized for the unique memory profile of that system”.

The raw reads generated by Illumina MySeq were uploaded to the Galaxy web platform to analyze the data. The reads had quality scores higher than 30, so the adapter sequences were trimmed before we performed the assembly with default parameters, obtaining 53,967 sequences in FASTA files, which were filtered by length with FASTA manipulation, a tool available in the Galaxy portal; next, we obtained 596 assembled sequences with lengths of 3000 to 12,000 nucleotides. The results of the analysis from the Galaxy platform were locally visualized with Mega 7 [19].

During the analysis, we found the sequences in both senses 5′→3′- and 3′→5′- for the same transcript, so in general, it was possible to determine the accuracy of the assemblies obtained. In many cases, we identified several sequences of the same protein with a different extension, so it was possible to make assemblies when the ends were correctly overlapped and allowed us to complete longer consensus sequences.

All transcripts were manually analyzed. The nucleotide sequences were in silico and translated according to the longest open-reading frames into protein sequences using the ExPASy translate tool [20]; the deduced amino acids sequences were compared with the BLAST program to search for homologous proteins reported for species in the National Center for Biotechnology Information (NCBI) (Bethesda, MD, USA) [21]. Homologous sequences of *P. clarkii* and other species were retrieved from GenBank [22]. We performed the alignment with ClustalW [23], and confirmed conserved domains, families, and functional sites by consulting the Prosite database [24], with protein function revised in the UniprotKB/Swiss-Prot database [25]. The identification of RNA transcripts in this study were edited and deposited in the NCBI. 

## 3. Results

### 3.1. Transcriptional Identification of Related Proteins in the Immune System of the Crayfish

We analyzed 596 transcriptomic sequences of 3000 to 12,000 nucleotides. During the analysis, we identified 80 mRNA molecules that were related to the immune system of decapods and arthropods. The information obtained for each sequence was used to integrate six tables according to the different known metabolic pathways of the crayfish immune system [26,27,28]. Additionally, we included transcripts that were involved in hematopoiesis [29] and endocytic routes [30].

The tables are organized as follows: the first column shows the name of the protein deduced from the transcriptome obtained from the crayfish *P. clarkii.* The second column indicates the number of nucleotides of the sequence reported by us, the number of amino acids (aa) deduced in silico from the sequence of *P. clarkii* with the best match with other species, and the accession numbers assigned by GenBank are included in the third and fourth columns, respectively. For all the sequences, we verified the existence of preserved domain profiles in the Prosite database, and correlated the function described for these proteins in the UNIPROT database; in the last column of each table, the functional activity determined from this database is briefly included for each protein.

The new sequences we identified in the abdominal nervous cord transcriptome of *P. clarkii* had 100% and 99% similarity with six sequences previously reported in this species; these results indicate the reliability of the results presented in this work. We found another three sequences that presented the highest homology with proteins that have already been reported for this crayfish, but with similarities of 44%, 48%, and 88%. The other 74 sequences in this text have not been previously reported in the GenBank database for the crayfish *P. clarkii*.

In Tables 1 to 6, we describe the homology of 75–99% with sequences reported for some other decapods of the species *Pacifastacus leniusculus* and *Litopenaus vannamei*, where there has been progress in the characterization of the molecular mechanisms of their immune system [31,32]. For comparison, we have included the 80 transcriptomic sequences described in this work as Supplementary Material S1. The similarity of the *Procambarus clarkii*’s sequences with species such as *Hyalella azteca* are highly variable (37–93%); this small amphipod crustacean is one of the few crustaceans that has a sequenced genome and an increasing list of proteins with annotation; this has allowed us to identify homologous proteins in *P. clarkii* as a result of scarce sequences with annotation in other crustaceans. Some of the sequences reported here showed a lower similarity with crustaceans such as the *Caridean hydrothermal* vent shrimp *Rimicaris aff. Exoculata* (36%) as well as oysters, insects, and a mammal (21% and 27%). It should be noted that in these latter cases, although it had a low percentage of similarity with animals that are more phylogenetically distant to the crayfish, this is because up to the date of our analysis, there had been no available sequences for crustaceans in the GenBank database to make comparisons; however, the family signatures and conserved domains allowed us to complete the annotation.

### 3.2. Components Identified Related to the Coagulation and Melanization Immune Pathway

Table 1 includes 16 components that participate in the coagulation and melanization mediated by the prophenoloxidase-activating system (proPO) that has been characterized in several species of crustaceans [33,34,35,36]. It can be seen in this table that we identified the precursor of the receptor beta-1,3-glucan-binding protein, several enzymes, and regulators evolved in this system [37]. Of this group of transcripts that we identified in *P. clarkii*, only prophenoloxidase had been previously reported for this species in GenBank [38]. We obtained a sequence of 459 aa that showed a similarity of 88% with the sequence of 627 aa previously reported, and it may be that this sequence is the second prophenoloxidase as it has been found in the other two decapods: *Penaeus monodon* and *Litopenaeus vannamei* [39,40,41,42].

The transglutaminase sequence deduced from the *P. clarkii* mRNA exhibited the highest homology with the transglutaminase of *Pacifastacus leniusculus*, which has been cloned and extensively characterized [43]. It also has a homology greater than 70% with the sequences reported in several shrimp and crab species [44]. Table 1 also includes some regulators such as pacifastin, and fortunately we found both heavy and light chains, previously characterized in the hemolymph of the crayfish *Pacifastacus leniusculus* [45]. It is important to highlight that in this group, we also included alpha-2-macroglobulin because its modulating action was recently characterized in the proPO system of the shrimp *Litopenaeus vannamei* [46]. Hall et al. characterized the clotting protein precursor in *Pacifastacus leniusculus* with 1721 aa [47]. The sequence of *P. clarkii* has a 73% similarity with the prawn *Pacifastacus leniusculus*, 39% similarity with the kuruma prawn *Marsupenaeus japonicus*, and 38% with the white shrimp *Litopenaeus vannamei* [48]. These results are important as it makes it evident that even among crustaceans, there are particularities that may require further analysis

### 3.3. Pattern Recognition Receptor and Apoptosis Components Identified in Crayfish

A group of several sequences described as pattern recognition receptors (PRR) and some elements involved in apoptosis are described in Table 2. Out of the 15 transcripts in this group, seven sequences were heat shock proteins (HSPs); only one sequence identified for us in *P. clarkii* had been previously reported for this species, with the other six sequences having a high similarity with several species of Decapoda and with *Philodinia roseola*, a microscopic animal component of zooplankton found in inland waters. For the other eight sequences, only calmodulin has been annotated in GenBank for *P. clarkii*, which was isolated from the axial abdominal crayfish muscle [49]; there is only one aa of difference with the sequence identified by us in the abdominal cord.

### 3.4. Complement Pathway Components Identified in Crayfish

We also identified 18 transcripts that participate in the complement pathway (Table 3). In this group, the activating transcription factor 4 is the only gene that has been previously reported for *P. clarkii*; the transcript that we identified recently had a 99% similarity with the one previously studied [50,51]. The remaining sequences shown in this table have been not identified in *P. clarkii* up to the date of the manuscript being sent; even for this group, several sequences have only been characterized in insects as can be seen in Table 3, thus the sequences that we report here are the first references for decapods.

### 3.5. Endocytic Route Components Identified in Crayfish

We describe in Table 4 several components of the endocytic pathway and other proteins involved in controlling pathogenic agents once they have been internalized to host cells through lysis processes. We also included some transcripts that participate in the endocytic pathway characterized during viral infection in *Cherax quadricarinatus* [30]. The seven transcripts included in Table 4 present a high similarity with different crustaceans, but we did not find homologous sequences for *P. clarkii*.

### 3.6. Anti-Microbial Peptides Identified in Crayfish

In the following table, we included five sequences that we identified in the transcriptome of the abdominal cord of *P. clarkii* (Table 5); four of them have been previously reported and have high homology, such as Crustin 2, and we identified both sequences previously reported.

There is good evidence that the Toll4 receptor is a regulator of the expression of antimicrobial peptides termed as the anti-lipopolysaccharide factor (ALF). For the *P. clarkii* species, 11 sequences of ALF, identified as ALF1 to ALF11, have been reported [53]; we identified a sequence composed of 438 aa that represents only a 44% similarity with ALF 10, which is a peptide of 242 aa length, and we reported this new transcript as ALF12.

The lysozyme sequence identified by our group had a 48% similarity with the sequence previously reported, which suggests that we have identified a new isoform of the known sequence. 

### 3.7. Toll, Immune Deficiency Pathway, and Ubiquitin-Proteasome System Components Identified in Crayfish

In Table 6, we list 19 transcripts involved in the Toll and Imd pathway that we identified in *P. clarkii*, of which only two sequences have been registered in GenBank for this species. One of these sequences represents a 99% similarity with the NF-kappa B transcription factor ID AGZ84432.1; nevertheless, the sequence obtained by our group adds 33 aa to this existing sequence. The other sequence, the ubiquitin-conjugating enzyme E2 UBC9 had a 99% homology and was previously characterized in *P. clarkii* during a viral infection [54]. This table also includes 12 elements of the ubiquitin-proteasome system that we identified in *Procambarus clarkii* and annotated in GenBank; this system is crucial for the maintenance of cellular integrity and may play an important role in stress resistance and shrimp pathogen defense [55]. In the abdominal nervous cord of *P. clarkii*, several subunits of proteasomes that integrate the 26S complex as demonstrated in humans have been identified [56], and five sequences of this system have only been reported in insects.

## 4. Discussion

Characteristically, invertebrates only possess mechanisms of innate immunity, both cellular and humoral, that are apparently activated depending on the pathogens involved [57,58]. The mechanisms of innate humoral immunity include coagulation of the hemolymph, melanization, lysosome synthesis, and antimicrobial peptides, reactive oxygen species, and reactive nitrogen species generation as well as proteolytic enzymes synthesis. In general, these defense mechanisms are triggered by the activation of the pathogen recognition receptors (PRR) [59,60].

Unlike other decapod species, basic knowledge of the molecular characteristics of the immune system of *Procambarus clarkii* is scarce. In the present work, we identified 80 abdominal cord transcripts related to the immune system pathways of various invertebrates; only six of these represented a match of 99–100% with the previously reported sequences in GenBank, with the other 74 sequences being reported for the first time in this species.

Shen et al. provided a comparative analysis of the transcriptome of muscle, ovary, testis, and hepatopancreas in *P. clarkii* [61]. Their investigation had only 330 ESTs and 547 nucleotide sequences as a background; these had been deposited in GenBank up until July 2014 for this species, and the authors noted that very few genetic markers had been discovered for *P. clarkii* until that date, although in recent years, the transcriptomes of various organs have been published, such as microRNAs potentially related to immunity [62], the lymph organ [63], testis, and ovary [64]. The number of sequences currently published in GenBank is 801 ESTs; hence, there is a need to carry out assembly and bioinformatic analysis of these sequences to provide easy access to all researchers and advance study at the molecular level of any organism.

We found a high similarity when comparing our sequences of *P. clarkii* with other sequences previously reported by different authors, allowing us to infer the accuracy of the sequences obtained by NGS with the Illumina protocol that we used. Thus, we consider that the sequences we are reporting on are sufficiently accurate and reliable to be considered as a starting point for functional studies.

It can also be observed that in a large proportion of the transcripts that we identified, the best match was with a crustacean amphipod, *Hyalella azteca*. This result may be because up to the date of preparation of this work (June–July 2018), there had been no reported sequences for any other decapod species, such as crabs, lobsters, and prawns. So far, we have reported some elements that participate in the immune system by identifying all sequences in the abdominal ganglionic cord transcriptome; it is likely that the hemolymph present in the interstices allowed us to identify various elements generally described in the hemocytes.

It is true that many sequences have been identified in the databases and confirmation of functional actions is required. However, the analysis of the transcriptome allows for the identification of highly conserved sequences through different arthropod kinds and increases our knowledge regarding diverse aspects. For example, in the heat shock protein 70 (Hsp70) sequence with accession number MG910465 (Table 3) identified during this work, the signature domain endoplasmic reticulum targeting is present, a characteristic sequence of proteins that permanently resides in the lumen of the endoplasmic reticulum (ER) [65,66].

In other cases, it allows us to complement existing sequences, such as in the sequence for the nuclear factor NF-kappa B; in GenBank, there is a sequence of 581 aa for *P. clarkii* [67] with access number AGZ84432.1. The sequence that we reported had 614 aa, with an addition of 33 aa to the existing sequence, with the additional amino acids making up the profile identified in Expasite Prosite as PS50322 GLN:RICH glutamine-rich region profile.

The present analysis even helped us to specify point mutations. In *Procambarus clarkii*, we identified two Crustin 2 sequences that differed only in two amino acids at positions 9 and 49. Interestingly, in GenBank, there were two sequences for Crustin 2 reported by two different authors that differed in these same positions; therefore, each of the Crustin 2 sequences that we identified had a 100% match with each sequence; one of them with access number AEB54630.1 [68] and the sequence with ID ACY64752.1 [69]. This background suggests that they are isoforms for the same antimicrobial peptide Crustin 2, and that both isoforms probably coexist in the abdominal ganglion. 

Transcriptome analysis rapidly generates the addition of new sequences for important protein families, such as in the case of lectins. There was one 125 sequence of C-type lectin reported in crustaceans in GenBank, seven of them in *P. clarkii*. In this work, we report two new sequences expressed in the abdominal ganglion cord.

GenBank is a public database of nucleotide and amino acid sequences, and is the most important tool globally because it makes sequences accessible to those who do not have extensive knowledge of bioinformatics and avoids repetition of sequences that have already been described. It further allows the design of some tools directed at the molecular level in highly specific studies, identifying in silico the functional domains of interest, and even establishing probable cellular locations.

It is essential to analyze sequences generated from NGS technologies to publish predicted proteins more quickly in databases as accessible as the NCBI. We expect that the development of the present work will allow the accessibility and diffusion of the recently obtained data and influence the development of new strategies for the study and control of illnesses of the crayfish *Procambarus clarkii,* such as the RNA interference (RNAi), one potential mechanism considered for the development of strategies for the treatment and control of the disease of farmed aquatic animals. The RNAi is based on their sequence specific ability to silence target genes, therefore, knowledge at a molecular level of the elements involved in the host-pathogen interaction is crucial to develop efficient strategies [70,71].

## 5. Conclusions

In this work, we identified and characterized for the first time 74 sequences in the transcriptome of the abdominal ganglion cord of the crayfish *Procambarus clarkii*. We used bioinformatic analysis to identify and establish the presence of the domain profile and homology of our sequences with sequences already existing in the GenBank database. All these new sequences have functional activity in the immune system of several invertebrates, and most of these transcriptomic sequences constitute the first reference of components that participate in the functional immune system pathways of the crayfish *P. clarkii*. We have reported these new sequences in the GenBank database from NCBI to make high-quality sequences available and facilitate their comparative phylogenetic analysis in future studies at the molecular level. In addition, from these sequences, functional studies can be carried out for this species, which has only been scarcely studied until now.

## Figures and Tables

**Table 1:** mRNAs identified related to the coagulation and melanization immune pathway in *Procambarus clarkii*.

Name	Nucleotides	Homology (aa)	Id GenBank	Function
Beta-1,3-glucan-binding protein precursor	10,743	*Astacus astacus* 3070/3607 (85%)	KY974279	Involved in recognition of invading microorganisms. Binds specifically to beta-1,3-glucan and activates the phenoloxidase cascade.
Transglutaminase	2178	*Pacifastacus leniusculus* 688/766 (90%)	MF385053	Enzyme of clotting system
Clotting protein precursor	5127	*Pacifastacus leniusculus* 1257/1725 (73%)	MG452709	Forms stable clots in the presence of calcium.
Laccase-10-like	2814	*Hyalella azteca* 262/656 (40%)	MG452692	Multicopper oxidases, oxidizes many different types of phenols and diamines.
Laccase-1-like	2043	*Bombus impatiens* 280/645 (43%)	MG452693	Laccases act on phenols and similar molecules
Alpha-2-macroglobulin-like isoform 3	4659	*Pacifastacus leniusculus* 1312/1606 (82%)	MG452688	A2M-like proteins can inhibit all four classes of proteinases by a ‘trapping’ mechanism.
Fibrinogen	2031	*Daphnia magna* 121/223 (54%)	MG452685	Fibrinogen, the principal protein of vertebrate blood clotting.
Pacifastin heavy chain precursor	3897	*Pacifastacus leniusculus* 805/917 (88%)	MG452696	Participates in the control of the prophenoloxidase (ProPo) system
Pacifastin light chain-like serine proteinase inhibitor	3405	*Litopenaeus vannamei* 249/637 (39%)	MG452697	
Masquerade -like serine proteinase (cMasII)	1110	*Pacifastacus leniusculus* 320/369 (87%)	MG452712	Participates in the ProPo system. The N-terminal region exhibited in vitro antimicrobial activity against Gram-positive bacteria
Hemocytin-like	1455	*Hyalella azteca* 151/374 (40%)	MG452718	Adhesive protein and relates to hemostasis or encapsulation of foreign substances for self-defense
Prophenoloxidase	1377	*Procambarus clarkii* 220/249 (88%)	MH156427	The terminal component of the so-called proPO activating system. This is a copper-containing oxidase that functions in the formation of pigments such as melanins and other polyphenolic compounds.
Flocculation protein FLO11-like	1857	*Hyalella azteca* 142/300 (40%)	MG976887	Cell wall protein that participates in adhesive cell-cell interactions during yeast flocculation, a reversible, asexual, and Ca^2+^-dependent process in which cells adhere to form aggregates (flocs) consisting of thousands of cells.
Tyrosine-protein kinase Fer	2619	*Hyalella azteca* 673/909 (74%)	KY974273	Plays a role in leukocyte recruitment and diapedesis in response to bacterial lipopolysaccharide (LPS).
Techylectin-5B-like	858	*Hyalella azteca]* 166/245 (68%)	MG976886	Lectin involved in innate immunity. Agglutinates all types of human erythrocytes, Gram-positive, and -negative bacteria. Has a stronger agglutinating activity towards Gram-negative bacteria than -positive bacteria. Specifically recognizes acetyl group-containing substances on agglutinated cells.
Integrin	2430	*Litopenaeus vannamei* 671/811 (83%)	MF140473	Integrins are cell adhesion molecules that mediate cell extracellular matrix and cell-cell interactions. They contain both alpha and beta subunits.

**Table 2:** Pattern recognition receptor (PRR) and apoptosis components identified in crayfish.

Name	Nucleotides	Homology (aa)	Id GenBank	Function
Heat shock protein 70 kDa (hsp70)	1914	*Procambarus clarkii* 635/638 (99%)	MH156459	The organisms respond to heat shock or other environmental stresses by inducing synthesis of proteins collectively known as heat-shock proteins
Heat shock protein 70 kDa cognate 3	1962	*Cherax cainii* 627/654 (96%)	MG910465	
Heat shock protein 70 kDa isoform 1	1962	*Astacus astacus* 646/658 (98%)	MG910466	
Heat shock protein 70 kDa isoform 2	1971	*Philodina roseola* 554/607 (91%)	MG910467	
Heat shock protein 84 kDa	2208	*Philodina roseola* 707/737 (96%)	MG910468	
Heat shock protein 90 kDa	2190	*Cherax destructor* 708/740 (96%)	MG910469	
Small heat shock protein	552	*Cherax destructor* 178/184 (97%)	MG910470	Small heat shock proteins acting as chaperones that can protect other proteins against heat-induced denaturation and aggregation.
Glycogen synthase kinase 3 beta (GSK3beta)	1230	*Litopenaeus vannamei* 403/410 (98%)	KY974307	Probably regulates NF-kappa-B (NFKB1) at the transcriptional level and is required for the NF-kappa-B-mediated anti-apoptotic response to TNF-alpha (TNF/TNFA).
Tax1 binding protein	2838	*Scylla serrata* 418/1027 (41%)	MG976888	Inhibits TNF-induced apoptosis by mediating TNFAIP3 anti-apoptotic activity. Degraded by caspase-3-like family proteins upon TNF-induced apoptosis. May also play a role in the pro-inflammatory cytokine IL-1 signaling cascade.
Tumor necrosis factor (TNF)	1524	*Metanephrops japonicus* 248/516 (48%)	MG452707	TNF-related apoptosis inducing ligand (TRAIL)
Tumor Necrosis Factor Super Family (TNFSF)	1476	*Litopenaeus vannamei* 258/496 (52%)	MG452708	TNF-related apoptosis inducing ligand (TRAIL) Membrane receptor involved in phagocytosis by macrophages and astrocytes of apoptotic cells. Receptor for C1q, an eat-me signal, that binds phosphatidylserine expressed on the surface of apoptotic cells
Multiple epidermal growth factor-like domains protein 10-like	917	*Saccoglossus kowalevskii* 62/232 (27%)	MH156433	TNF-related apoptosis inducing ligand (TRAIL) Membrane receptor involved in phagocytosis by macrophages and astrocytes of apoptotic cells. Receptor for C1q, an eat-me signal, that binds phosphatidylserine expressed on the surface of apoptotic cells. Regulates osteoblast maturation by controlling TGF-beta bioavailability and calibrating TGF-beta and bone morphogenetic protein (BMP) levels, respectively.
Fibrillin 2	5139	*Hyalella azteca* 1241/1725 (72%)	MG910478	
Calmodulin	447	*Procambarus clarkii* 148/149 (99%)	MG910474	Calmodulin mediates control of many enzymes, ion channels, aquaporins, and other proteins by Ca^2+^.
Stabilin 2	2010	*Lingula anatine* 167/493 (34%)	MH492359	Phosphatidylserine receptor that enhances the engulfment of apoptotic cells. Binds to both Gram-positive and -negative bacteria and may play a role in defense against bacterial infection.

**Table 3:** Complement pathway components identified in *Procambarus clarkii*.

Name	Nucleotides	Homology (aa)	Id GenBank	Function
C-type lectin	687	*Litopenaeus vannamei* 68/147 (46%)	MG452686	Protein domains homologous to the carbohydrate-recognition domains (CRDs) of the C-type lectins.
C-type lectin	930	*Portunus trituberculatus* 141/213 (66%)	MG452687	C-type lectin (CTL) or carbohydrate-recognition domain (CRD).
Galactose-specific lectin nattectin-like	567	*Hyalella azteca* 42/119 (35%)	MG910471	Exhibits hemagglutination activity (minimum hemagglutination concentration = 2.5 µg/well) in a calcium-independent fashion. Has remarkable pro-inflammatory activity, inducing neutrophil mobilization in mice.
Ras-related protein Rab-11A-like	642	*Penaeus monodon* 210/214 (98%)	KY974298	The small Guanosine Triphosphatase or GTPases Rab are key regulators of intracellular membrane trafficking. Regulates the recycling of receptor of Fc region of monomeric Ig G (FCGRT) to basolateral membranes.
Casein kinase I	987	*Hyalella azteca* 284/307 (93%)	MF062030	Probably involved in lymphocyte physiology.
Activating transcription factor 4	1293	*Procambarus clarkii* 430/431 (99%)	MG976885	ATF4 regulates the expression of genes involved in oxidative stress [51].
Dual oxidase 2-like	1407	*Hyalella azteca* 333/472 (71%)	MF688645	Generates hydrogen peroxide, required for the activity of Thyroid Peroxidase (TPO) and Lactoperoxidase (LPO) Plays a role in thyroid hormones synthesis and lactoperoxidase-mediated antimicrobial defense at the surface of mucosa.
Polypeptide N-acetilgalactosaminyltransferase 2	1659	*Zootermopsis nevadensis* 272/549 (50%)	MG910472	Probably involved in O-linked glycosylation of the immunoglobulin A1 (IgA1) hinge region
Angiopoietin-related protein 2-like	1446	*Hyalella azteca* 140/239 (59%)	MG452689	ANGPTL2 has a role also in angiogenesis, in tissue repair.
Thrombospondin-1	2859	*Zootermopsis nevadensis* 356/845 (42%)	MG452690	Adhesive glycoprotein that mediates cell-to-cell and cell-to-matrix interactions.
Angiopoietin-2-like	1983	*Hyalella azteca* 242/490 (49%)	MG452694	Binds to TEK/TIE2, competing for the ANGPT1 binding site, and modulating ANGPT1 signaling.
Wiskott-Aldrich Syndrome protein (WASp) family member 3 isoform X1	1175	*Zootermopsis nevadensis* 131/292 (45%)	MG452710	WASp is required for various functions in myeloid and lymphoid immune cells
Wiskott-Aldrich syndrome protein family member 3-like	1143	*Hyalella azteca* 163/243 (67%)	MG452711	
B-cell receptor-associated protein 31-like	690	*Hyalella azteca* 136/230 (59%)	MG452713	Functions as a chaperone protein.
B-cell differentiation antigen CD72	672	*Vicugna pacos* 51/192 (27%)	MG452715	Plays a role in B-cell proliferation and differentiation.
CD109 antigen-like	5160	*Crassostrea gigas* 148/706 (21%)	MG452716	Modulates negative TGFB1 signaling in keratinocytes.
Vascular endothelial growth factor 2	1173	*Litopenaeus vannamei* 172/334 (51%)	MG452717	Growth factor active in angiogenesis, vasculogenesis, and endothelial cell growth.
Peroxidasin homolog	1392	*Hyalella azteca* 229/323 (71%)	MG910475	Plays a role in extracellular matrix consolidation, phagocytosis, and defense. Contains the IG-like domain profile.

**Table 4:** Endocytic route components identified in *Procambarus clarkii*.

Name	Nucleotides	Homology (aa)	Id GenBank	Function
Beta-Tubulin 1	1353	*Daphnia pulex* 403/451 (89%)	MG910477	Tubulin is the major constituents of microtubules. It binds two moles of GTP, one at an exchangeable site on the beta chain and one at a non-exchangeable site on the alpha chain.
Actin, cytoplasmic 1	1128	*P. tepidariorum* 360/376 (96%)	MG910479	Actins are highly conserved proteins that are involved in various types of cell motility and are ubiquitously expressed in all eukaryotic cells.
Clathrin	4206	*F. chinensis* 1383/1402 (99%)	KY974296	Participates in the internalization of viruses.
Gamma-aminobutyric acid receptor-associated protein GABARAP	357	*C. quadricarinatus* 117/119 (98%)	MG910480	Participates in the formation of autophagosomes.
Sortilin-related receptor-like	3348	*Hyalella azteca* 491/1176 (42%)	MF279130	Likely to be a multifunctional endocytic receptor that may be implicated in the uptake of lipoproteins and of proteases.
Cathepsin A	1458	*Eriocheir sinensis* 348/453 (77%)	MG452720	Enclosed within lysosomes, participates in innate immune system.
Endoplasmin	2355	*Penaeus monodon* 612/722 (85%)	MG452719	Molecular chaperone that functions in the processing and transport of secreted proteins.

**Table 5:** Anti-microbial peptides (AMPs) identified in *Procambarus clarkii*.

Name	Nucleotides	Homology (aa)	Id Genbank	Function
ALF12	1314	*Procambarus clarkii* 44/101 (44%)	MH538268	May bind to bacterial LPS and thus specifically inhibit LPS-mediated activation of hemolymph coagulation. It has a strong antibacterial effect, particularly on the growth of Gram-negative bacteria.
Crustin 2	330	*Procambarus clarkii* 110/110 (100%)	MG976884	Crustins act primarily against Gram-positive bacteria, although some have also been reported to kill Gram-negatives [52]
Crustin 2 isoform 1	267	*Procambarus clarkii* 89/89 (100%)	MH492354	
Lysozyme-like	456	*Procambarus clarkii* 71/149 (48%)	MG976883	Has bacteriolytic activity. May play a role in digestion and in host defense mechanisms against invading microbes
Lysosome-associated membrane glycoprotein 1-like	990	*Hyalella azteca* 92/245 (38%)	MG452714	LAMP are integral membrane proteins, specific to lysosomes, and their exact biological function is not yet clear.

**Table 6:** Toll, Immune deficiency pathway (Imd), and ubiquitin-proteasome system components identified in *Procambarus clarkii*.

Name	Nucleotides	Homology (aa)	Id GenBank	Function
Toll-like receptor 3	1902	*Hyalella azteca* 320/522 (61%)	MG452691	Toll type receptors recognize molecular patterns expressed by a broad spectrum of infectious agents.
Sequestosome-1	1014	*Lingula anatina* 83/225 (37%)	MG452705	May regulate the activation of NFKB1 by TNF-alpha, nerve growth factor (NGF), and interleukin-1. May regulate signaling cascades through ubiquitination.
Ubiquitin-activating enzyme E1	3114	*Eriocheir sinensis* 866/1014 (85%)	MG452698	Activates ubiquitin by first adenylating with Adenosine Triphosphate (ATP) its C-terminal glycine residue
Ubiquitin-conjugating enzyme E2 UBC9	480	*Procambarus clarkii* 159/160 (99%)	MG452699	Catalyzes covalent attachment of ubiquitin to target proteins.
Ubiquitin conjugating enzyme-3	495	*Eriocheir sinensis* 165/165 (100%)	MG452700	Accepts ubiquitin from an E2 ubiquitin-conjugating enzyme in the form of a thioester and then directly transfers ubiquitin to targeted substrates
E3 ubiquitin-protein ligase UBR5	1092	*Zootermopsis nevadensis* 283/382 (74%)	MG452701	
Ubiquitin-protein ligase E3A	1128	*Stegodyphus mimosarum* 275/376 (73%)	MG452702	
Ubiquitin carboxyl-terminal hydrolase 7-like	2631	*Hyalella azteca* 705/904 (78%)	MG452703	Deubiquitinating enzymes
Proteasome subunit beta type-3	615	*Hyalella azteca* 165/204 (81%)	MG910481	Associated with two 19S regulatory particles, forms the 26S proteasome and thus participates in the ATP-dependent degradation of ubiquitinated proteins.
26S Proteasome non-ATPase regulatory subunit 2-like	2166	*Hyalella azteca* 578/732 (79%)	MG910482	Component of the 26S proteasome, a multiprotein complex involved in the ATP-dependent degradation of ubiquitinated proteins.
26S Proteasome non-ATPase regulatory subunit 3-like	1497	*Camponotus floridanus* 330/504 (65%)	MG910483	
26S Proteasome non-ATPase regulatory subunit 4-like	1320	*Fopius arisanus* 258/440 (59%)	MG910484	
26S Protease regulatory subunit 7-like	510	*Hyalella azteca* 149/170 (88%)	MG910485	
Papain family cysteine protease	798	*Tetrahymena thermophila* SB210 138/269 (51%)	MH156429	Papain-like cysteine proteinase: plays a role in inhibition of interferon-activated JAK/STAT signal transduction by mediating ubiquitination and subsequent proteasomal degradation of host KPNA1.
Leucine-rich repeats and immunoglobulin-like domains protein 1	1428	*Hyalella azteca* 228/454 (50%)	MG976882	Acts as a negative feedback regulator of signaling by tyrosine receptor kinases through a mechanism that involves enhancement of receptor ubiquitination and accelerated intracellular degradation.
Large proline-rich protein BAG6	1086	*Zootermopsis nevadensis* 114/369 (31%)	MG976889	BAG6 is also required for selective ubiquitin-mediated degradation of defective nascent chain polypeptides by the proteasome. In this context, it may participate in production of antigenic peptides and play a role in antigen presentation in the immune response
Chitin binding-like protein	591	*Fenneropenaeus chinensis* 65/189 (34%)	MF688647	Binds chitin but does not hydrolyze it; has no detectable protease or staphylolytic activity.
NF-kB Transcription factor Relish	1842	*Procambarus clarkii* 580/581 (99%)	MG910473	The endpoint of a series of signal transduction events initiated by a vast array of stimuli related to many biological processes such as inflammation, immunity, differentiation, cell growth, tumorigenesis, and apoptosis.
Transforming growth factor-beta-induced protein ig-h3-like	1470	*Cryptotermes secundus* 143/492 (29%)	MG910476	Plays a role in cell adhesion. May play a role in cell-collagen interactions.

## Supplementary Materials

File S1: 80 transcriptomic sequences of *P. clarkii* described in column 1 of the Table 1, Table 2, Table 3, Table 4, Table 5 and Table 6. Available online at http://www.mdpi.com/2571-5135/7/3/26/s1.
